# Supplementary material for: Increased Renal Medullary NOX-4 in Female but Not Male Mice during the Early Phase of Type 1 Diabetes: Potential Role of ROS in Upregulation of TGF-β1 and Fibronectin in Collecting Duct Cells
Source: Antioxidants (Basel). 2023 Mar 16;12(3):729. doi: 10.3390/antiox12030729 (PMC10045926; doi:10.3390/antiox12030729)
Supplement: Supplementary file 1 [file antioxidants-12-00729-s001.zip › antioxidants-2267636-supplementary.pdf]

SUPPLEMENTARY MATERIALS

Increased Renal Medullary NOX-4 in Female but Not Male Mice during the Early Phase of Type 1 Diabetes: Potential Role of ROS in Upregulation of TGF-β1 and Fibronectin in Collecting Duct Cells

Felipe Casado-Barragán [1,†], Geraldine Lazcano-Páez [1,†], Paulina E. Larenas [1], Monserrat Aguirre-Delgadillo [1], Fernanda Olivares-Aravena [1], Daniela Witto-Oyarce [1], Camila Núñez-Allimant [1], Katherin Silva [1], Quynh My Nguyen [2], Pilar Cárdenas [1], Modar Kassan [3] and Alexis A. Gonzalez [1,*]

Figure S1. Blots used to quantify protein levels

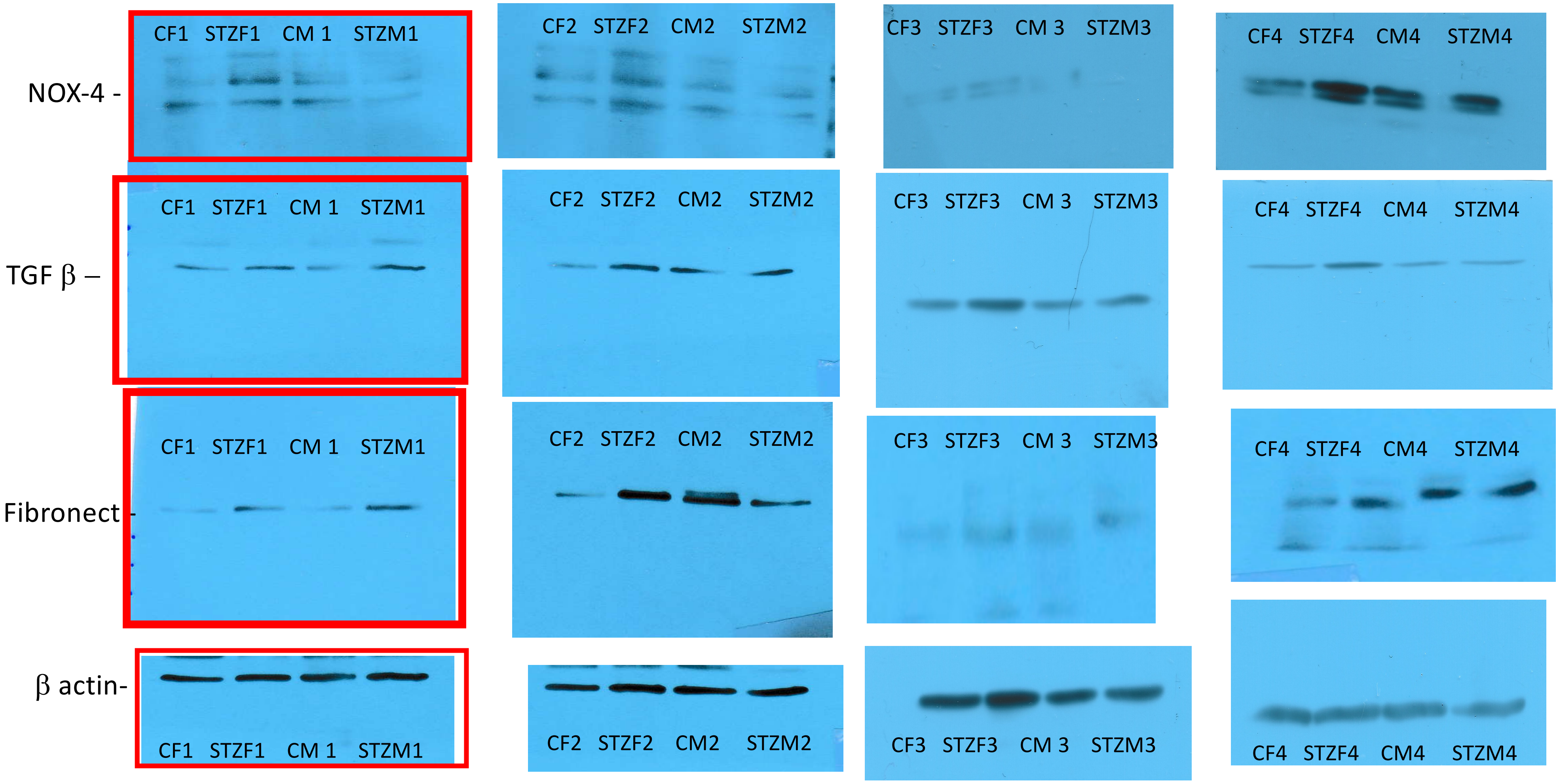

Replicates analyzed by immunoblots. CF= control females; STZF= females STZ; CM=control Males; STZM= males STZ

Figure S2. Gel identity and molecular weight

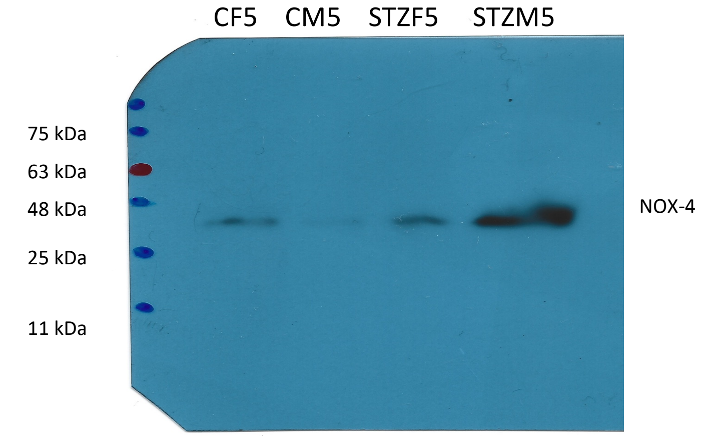

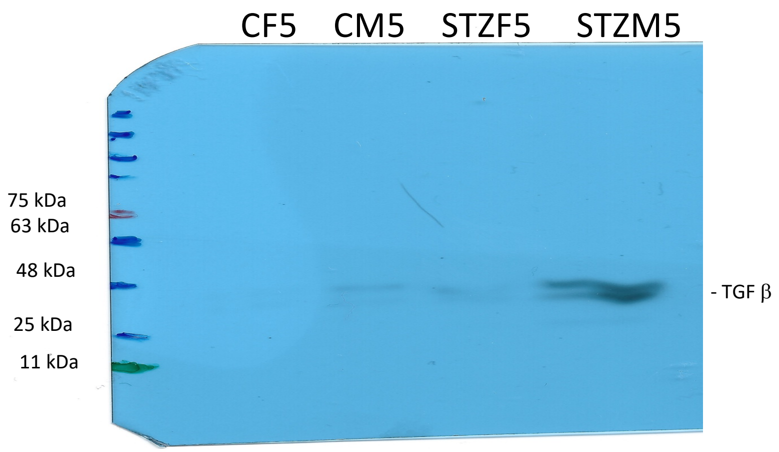

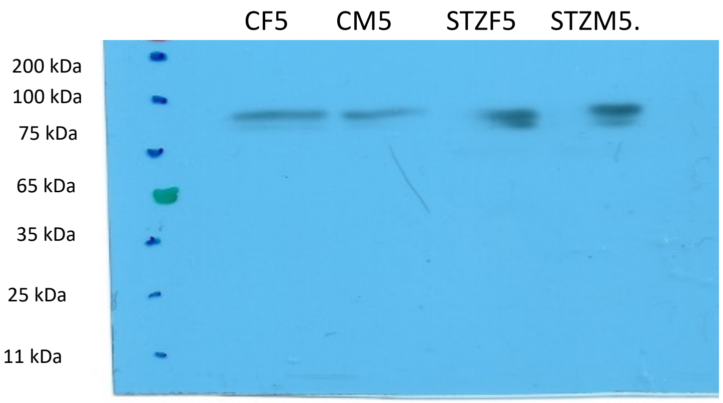

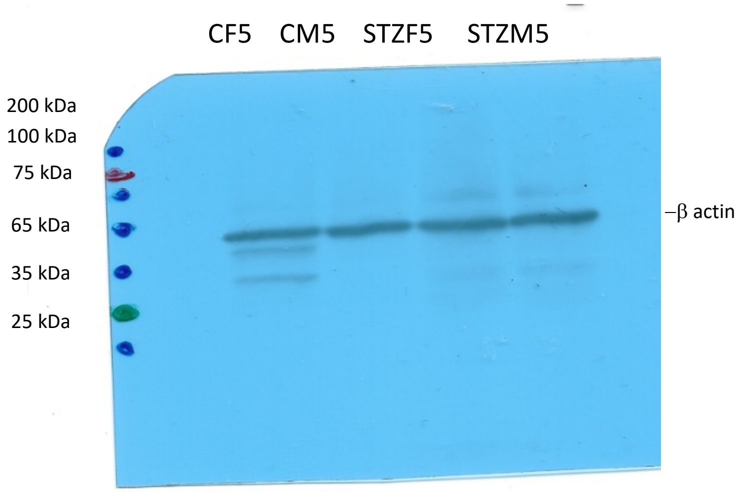

Molecular weight was only added in this gel to check band identity. Please consider that we were unable to load all the blots with molecular weight standards

Figure S3. IMCD cells from male and female showed same profile of expression in response to normal or high glucose

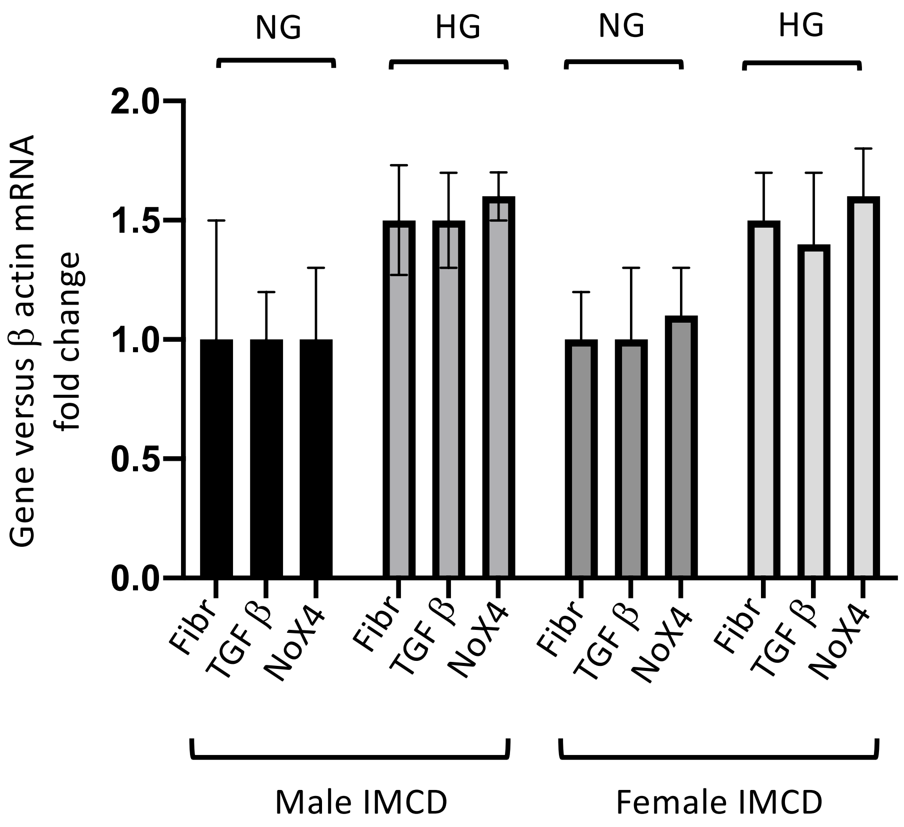

n=3

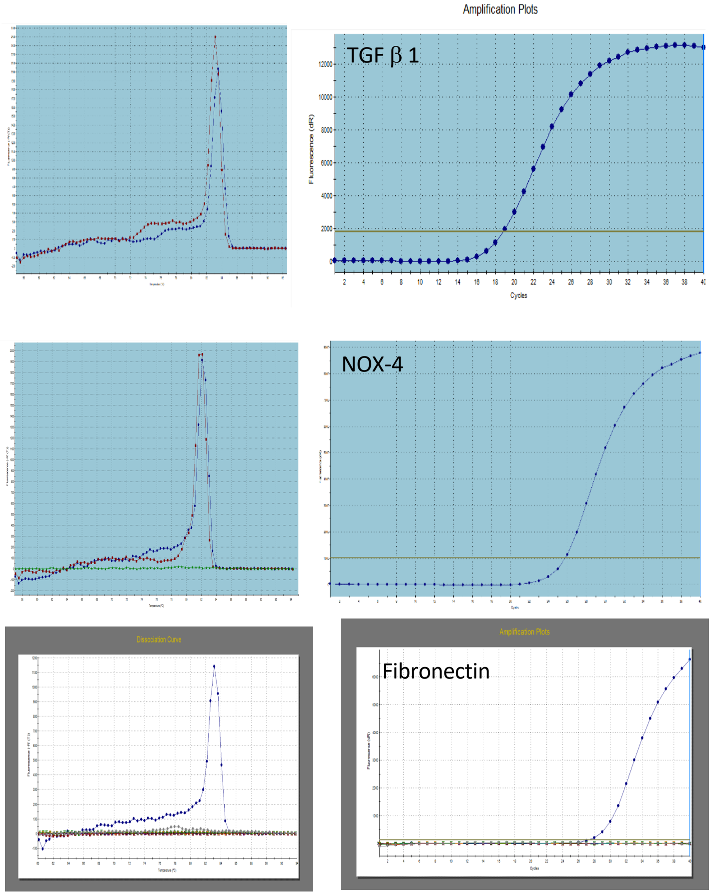

Cultured IMCD from male or female showed similar pattern of expression.

Figure S4 Gel identity and molecular weight for the representitve blot shown in Figure 6 B (NG1 vs HG1)

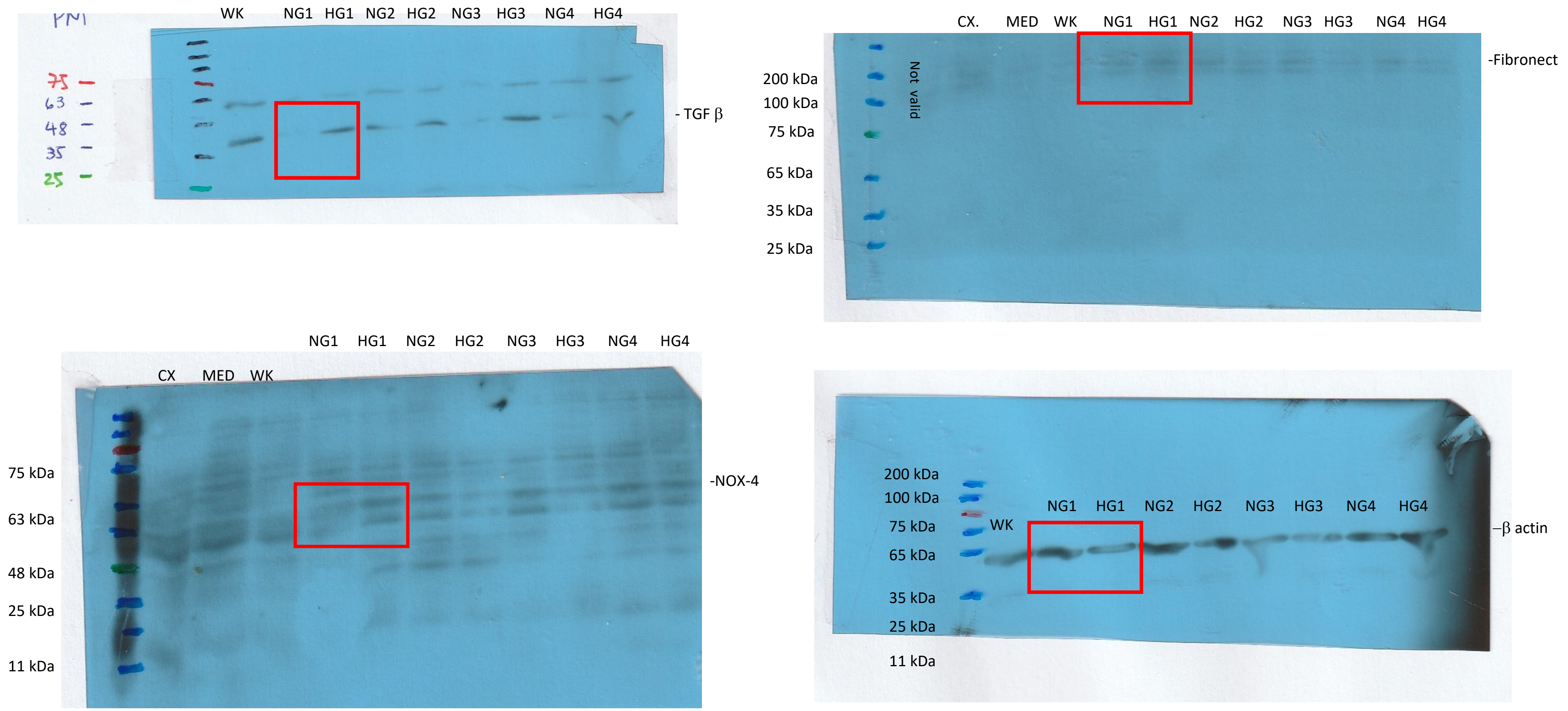

CX: cortex; MED: medulla; WK: whole kidney
n=4, IMCD cells icubated with NG= normo-glucose (5mM), HG= high-glucose (25 mM)

Figure S5. Control for osmolality was evaluated by using 25 mM of mannitol. Results showed no differences on the expression of profibrotic genes analysed.

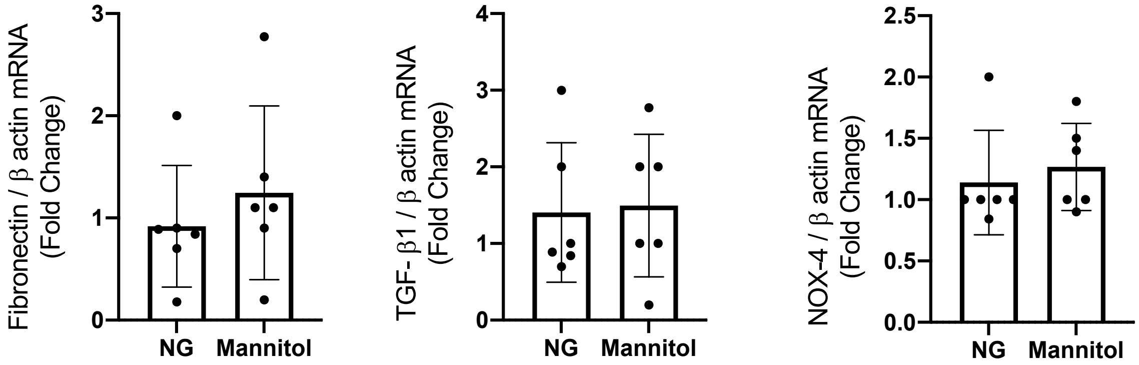

The possible effect of osmolar stress by adding glucose to IMCD cells by replacing by mannitol (25 mM) on the expression of profibrotic genes analyzed. No changes were seen, n=6.
